# Grass Carp Reovirus VP56 Allies VP4, Recruits, Blocks, and Degrades RIG-I to More Effectively Attenuate IFN Responses and Facilitate Viral Evasion

**DOI:** 10.1128/Spectrum.01000-21

**Published:** 2021-09-15

**Authors:** Hang Su, Zhiwei Liao, Chunrong Yang, Yongan Zhang, Jianguo Su

**Affiliations:** a Department of Aquatic Animal Medicine, College of Fisheries, Huazhong Agricultural University, Wuhan, China; b Laboratory for Marine Biology and Biotechnology, Pilot Qingdao National Laboratory for Marine Science and Technology, Qingdao, China; c Engineering Research Center of Green Development for Conventional Aquatic Biological Industry in the Yangtze River Economic Belt, Ministry of Education, Wuhan, China; d College of Veterinary Medicine, Huazhong Agricultural University, Wuhan, China; Wright State University

**Keywords:** grass carp reovirus, fibrin VP56, major outer capsid protein VP4, host-virus protein interaction, RIG-I, interferon, viral evasion

## Abstract

Grass carp reovirus (GCRV), the most virulent aquareovirus, causes epidemic hemorrhagic disease and tremendous economic loss in freshwater aquaculture industry. VP56, a putative fibrin inlaying the outer surface of GCRV-II and GCRV-III, is involved in cell attachment. In the present study, we found that VP56 localizes at the early endosome, lysosome, and endoplasmic reticulum, recruits the cytoplasmic viral RNA sensor retinoic acid-inducible gene I (RIG-I) and binds to it. The interaction between VP56 and RIG-I was detected by endogenous coimmunoprecipitation (co-IP), glutathione *S*-transferase (GST) pulldown, and subsequent liquid chromatography-tandem mass spectrometry (LC-MS/MS) and was then confirmed by traditional co-IPs and a novel far-red mNeptune-based bimolecular fluorescence complementation system. VP56 binds to the helicase domain of RIG-I. VP56 enhances K48-linked ubiquitination of RIG-I to degrade it by the proteasomal pathway. Thus, VP56 impedes the initial immune function of RIG-I by dual mechanisms (blockade and degradation) and attenuates signaling from RIG-I recognizing viral RNA, subsequently weakening downstream signaling transduction and interferon (IFN) responses. Accordingly, host antiviral effectors are reduced, and cytopathic effects are increased. These findings were corroborated by RNA sequencing (RNA-seq) and VP56 knockdown. Finally, we found that VP56 and the major outer capsid protein VP4 bind together in the cytosol to enhance the degradation of RIG-I and more efficiently facilitate viral replication. Collectively, the results indicated that VP56 allies VP4, recruits, blocks, and degrades RIG-I, thereby attenuating IFNs and antiviral effectors to facilitate viral evasion more effectively. This study reveals a virus attacking target and an escaping strategy from host antiviral immunity for GCRV and will help understand mechanisms of infection of reoviruses.

**IMPORTANCE** Grass carp reovirus (GCRV) fibrin VP56 and major outer capsid protein VP4 inlay and locate on the outer surface of GCRV-II and GCRV-III, which causes tremendous loss in grass carp and black carp industries. Fibrin is involved in cell attachment and plays an important role in reovirus infection. The present study identified the interaction proteins of VP56 and found that VP56 and VP4 bind to the different domains of the viral RNA sensor retinoic acid-inducible gene I (RIG-I) in grass carp to block RIG-I sensing of viral RNA and induce RIG-I degradation by the proteasomal pathway to attenuate signaling transduction, thereby suppressing interferons (IFNs) and antiviral effectors, facilitating viral replication. VP56 and VP4 bind together in the cytosol to more efficiently facilitate viral evasion. This study reveals a virus attacking a target and an escaping strategy from host antiviral immunity for GCRV and will be helpful in understanding the mechanisms of infection of reoviruses.

## INTRODUCTION

Grass carp reovirus (GCRV) seriously threatens production of grass carp (*Ctenopharyngodon idella*) and black carp (*Mylopharyngodon piceus*). It is a pathogenic agent with high virulence, leading to severe epidemic hemorrhagic disease outbreaks annually and tremendous mortality (up to 90%) (1). GCRV belongs to group C, *Aquareovirus* genus in the *Spinareovirinae* subfamily, *Reoviridae* family and is regarded as the most virulent virus in the *Aquareovirus* genus (2). The GCRV genome possesses 11 double-stranded RNA (dsRNA) segments (termed as S1 to S11) that encode 13 proteins, including 7 structural proteins and 6 nonstructural proteins (3, 4), encased in a double-layered icosahedral capsid shell (5). According to genomic and biological characteristics, over 30 GCRV strains are currently reported and classified into three types, which are respectively represented by strain GCRV-873 (type I), GCRV-HZ08 (type II), and GCRV104 (type III), with less than 20% amino acid sequence similarity between types, resulting in diverse encoded proteins (6). Type II GCRV (GCRV-II) has the highest prevalence and virulence among the three types and closer homology with mammalian orthoreovirus than other known species of aquareovirus based on phylogenetic analysis (7). Segment 7 of GCRV-II (strain 097) with a length of 1,560 bp encodes a fiber protein of approximately 56 kDa (defined as VP56) (8). Fiber protein is acknowledged to play a crucial role in the infection process of reovirus attaching to the surface of host cells (9, 10). The fibrin VP56 exists in GCRV-II and GCRV-III (9), but the attachment mechanism of GCRV is not certain to date. The functions of VP56 when the virus particle attaches to the host cell and after entry into the cell membrane are still unclear. Although several reports have claimed to understand part of the function of GCRV fiber protein, further research is needed (11).

Following pathogen invasion, innate immunity senses microbe-associated molecular patterns (MAMPs) by pattern recognition receptors (PRRs) to initiate immune responses. Retinoic acid-inducible gene I (RIG-I)-like receptors (RLRs) are a crucial family of cytoplasmic PRRs sensing viral RNA (12). The RLR family contains three members: RIG-I, melanoma differentiation-associated gene 5 (MDA5), and laboratory of genetics and physiology 2 (LGP2). RIG-I mainly recognizes RNAs with a 5′-triphosphate (PPP) group or short dsRNA of a wide variety of RNA or DNA viruses (13). Upon viral infection, the repressor domain (RD) recognizes the 5′-PPP extremity of the blunt-end base-paired RNA, and the helicase domain (DExD box helicase/ATPase domain [DExD/H]) binds to the sugar-phosphate backbone of duplexed RNA, resulting in the release of caspase-associated and recruitment domains (CARDs) (12). The CARDs of RIG-I physically interact with the CARD of interferon-β (IFN-β) promoter stimulator 1 (IPS-1), the adaptor protein of RLRs, to activate the downstream signaling cascade (14, 15). This activates stimulator of IFN genes (STING) (also known as MITA, ERIS, and MPYS) and TANK-binding kinase 1 (TBK1), leading to the phosphorylation of interferon regulatory factor (IRF) 3/7, which is translocated to the nucleus (16, 17) where they activate IFN gene transcription by binding to interferon-sensitive response element (ISRE) motifs present in IFN promoters (18). Fish also have a similar functional RLR pathway (19). Fish RIG-I and MDA5 intensively trigger IFN production (20–22). IRF3 and STING can be phosphorylated by TBK1, and they display a powerful capacity to activate IFN responses (23–25).

After millions of years of coevolution, along with genome mutations, gene recombination, and rearrangements, viruses have evolved mechanisms to escape host immune responses (26). For RNA viruses, their evolution or mutation is particularly rapid, so the immune escape mechanisms of RNA viruses are relatively advanced and have extremely high research value (27). Tegument protein UL37 of herpes simplex virus 1 (HSV-1) promotes RIG-I deamidation to inhibit viral dsRNA-triggered RIG-I activation (28). Nonetheless, there are few studies indicating the function of VP56 in the process of GCRV infection and its immune regulation mechanism related to RIG-I and the downstream IFN response (11). In the present study, we found that VP56 can bind to the RIG-I helicase domain. Furthermore, VP56 degrades RIG-I via a K48-linked ubiquitination-mediated proteasomal pathway to inhibit RIG-I-regulated IFN antiviral responses, resulting in GCRV replication and infection enhancement and viral evasion. In addition, VP56 binds to the GCRV major outer capsid protein VP4 in the cytosol, synergistically enhancing the degradation of RIG-I and more efficiently facilitating viral evasion. The present results reveal a viral invasion target and viral evasion strategy developed by aquareovirus involving the negative regulation of RIG-I. This study provides insights into the functions of viral fibrin and the viral escaping strategy and infection mechanisms of reoviruses.

## RESULTS

### VP56 binds to the helicase domain of RIG-I.

**FIG 1:**
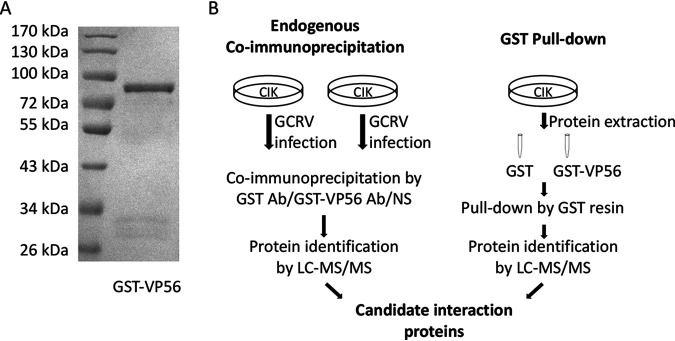
Schematic diagram of endogenous co-IP and GST pulldown. (A) Purified recombinant protein GST-VP56 was checked by SDS-PAGE. (B) Workflow on exploring interaction proteins with VP56 by endogenous co-IP and GST pulldown. Left, detecting VP56 interacting proteins via co-IP and LC-MS/MS. CIK cells were infected with GCRV for 24 h, and co-IP with GST monoclonal Ab, GST-VP56 purified polyclonal Ab, or negative serum (NS) was performed. LC-MS/MS analysis was performed on a Q Exactive mass spectrometer. Right, detecting VP56 interacting proteins via GST pulldown and LC-MS/MS. CIK proteins were exacted from CIK cells and incubated with purified recombinant GST or GST-VP56 protein. Pulldown was performed with GST resin, and the elution was examined by LC-MS/MS on a Q Exactive mass spectrometer. Candidate interaction proteins were obtained by analyzing these LC-MS/MS results. Triplicate independent experiments were performed with three biological replicates.

To research the molecular action of VP56 during GCRV infection, endogenous coimmunoprecipitation (co-IP), glutathione *S*-transferase (GST) pulldown, and subsequent liquid chromatography-tandem mass spectrometry (LC-MS/MS) were performed (Fig. 1). Recombinant GST-VP56 fusion protein was expressed in Escherichia coli BL21(DE3) pLysS cells and purified by affinity chromatography (Fig. 1A). The polyclonal antibodies (Abs) of GST-VP56 and GST were prepared by inoculating New Zealand White rabbits and were purified by antigen-antibody affinity chromatography, respectively. For endogenous co-IP, *C. idella* kidney (CIK) cells were infected with GCRV, and a co-IP was performed with VP56 polyclonal Ab, GST Ab, and negative serum, respectively, followed by LC-MS/MS (Fig. 1B, left). For GST pulldown, CIK cell proteins were extracted and incubated with VP56 protein for binding, while GST protein was used as a control. GST resin was then added into the complex. After washing and elution, the eluted protein mixture was analyzed by LC-MS/MS (Fig. 1B, right). Summarizing the LC-MS/MS results, several interactive proteins were identified from the proteomic data set, of which 77 candidate proteins matched at least two peptide fragments in a protein sequence (Table S1 in the supplemental material). Among the candidate interactive proteins, RIG-I was noticed.

To understand the molecular action of fibrin VP56 during GCRV infection, subcellular localization of VP56 was detected by confocal microscopy (Fig. S1). Fathead minnow (FHM) cells were cotransfected with VP56-green fluorescent protein (GFP) fusion vector and relevant organelle protein markers followed by 4′,6-diamidino-2-phenylindole (DAPI) staining for the cell nucleus. As shown in Fig. S1, appearances of yellow signals indicating overlapping of green and red were observed in cells cotransfected with VP56 and RAB5, LAMP2, and GRP78, which are the marker proteins of early the endosome, lysosome, and endoplasmic reticulum (ER), respectively. These results demonstrated that VP56 localizes at the early endosome (Fig. S1A), lysosome (Fig. S1C), and ER (Fig. S1D) but not the late endosome (Fig. S1B). Colocalizations of VP56 with cytoplasmic organelles imply the possible distribution of VP56 after virus uncoating in the cytoplasm.

**FIG 2:**
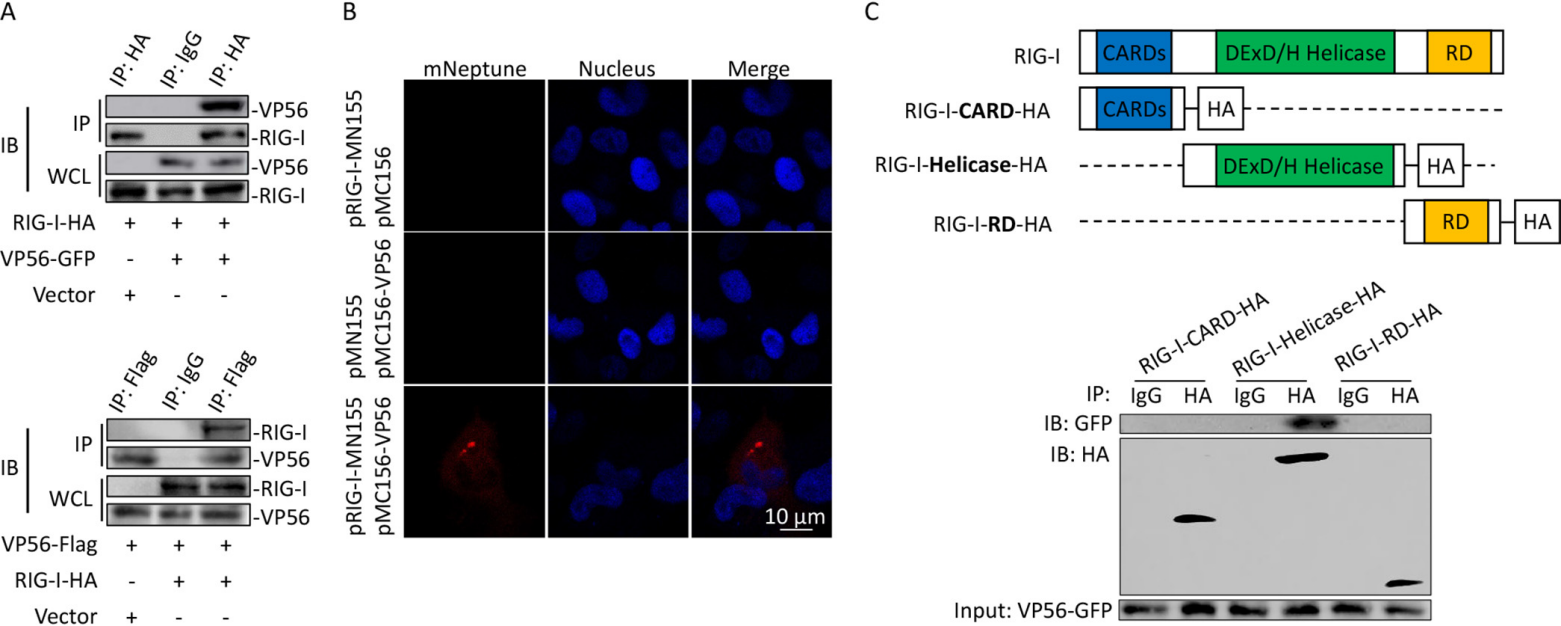
Verification of the interaction between VP56 and the helicase domain of RIG-I. (A) Verification of the interaction between VP56 and RIG-I by traditional co-IPs. Top, FHM cells were cotransfected with VP56-GFP/vector and RIG-I-HA for 48 h. Co-IP was performed with anti-HA monoclonal Ab and mouse IgG (control) and immunoblotting with the corresponding Abs. Bottom, FHM cells were cotransfected with RIG-I-HA/vector and VP56-Flag for 48 h. Co-IP was performed with anti-Flag monoclonal Ab and mouse IgG (control) and immunoblotting with the corresponding Abs. IB, immunoblot; WCL, whole-cell lysate. (B) Imaging of the VP56-RIG-I interaction by far-red mNeptune-based BiFC. Corresponding vectors were transfected alone or cotransfected into CIK cells under normal conditions. In the BiFC system, the fluorescence of the mNeptune channel was red, and the nucleus was stained with DAPI. Images were acquired using confocal microscopy under a 40× lens objective. Appearance of red fluorescence represents the positive observation. BiFC experiments were repeated in triplicate, and the images were selected and cropped to show the positive results. (C) VP56 interacts with the RIG-I-DExD/H helicase domain. FHM cells were cotransfected with 4 μg of VP56-GFP and 4 μg of RIG-I-CARD-HA or RIG-I-helicase-HA or RIG-I-RD-HA for 24 h in 10-cm^2^ dishes. Co-IP was performed using HA Ab, and mouse IgG was used as a control. IPs were analyzed by immunoblotting with anti-HA and anti-GFP, respectively. Expression of VP56-GFP (input) was examined with GFP Ab. All the co-IP and BiFC assays were repeated independently at least three times.

The direct interaction between VP56 and RIG-I was further confirmed by traditional co-IPs (Fig. 2A) and far-red mNeptune-based bimolecular fluorescence complementation (BiFC) (Fig. 2B). Positive bands were observed in both co-IP results conducted by RIG-I pulling down of VP56 and VP56 pulling down of RIG-I via tag Abs (Fig. 2A). According to the BiFC results, red fluorescence of mNeptune was not detected in single VP56 or RIG-I proteins overexpressed in cells. When CIK cells were transfected with both VP56 and RIG-I plasmids linked with each terminal of the mNeptune protein, these two fusion proteins present closely and send out red fluorescence signal (Fig. 2B). To further find out the specific interaction domain of RIG-I, a co-IP was performed, and the results indicated that the RIG-I helicase domain could interact with VP56 (Fig. 2C). Therefore, the interaction between VP56 and RIG-I was proven, and the binding site was found to be the helicase domain of RIG-I.

### VP56 enhances K48-linked ubiquitination to degrade RIG-I by the proteasomal pathway.

**FIG 3:**
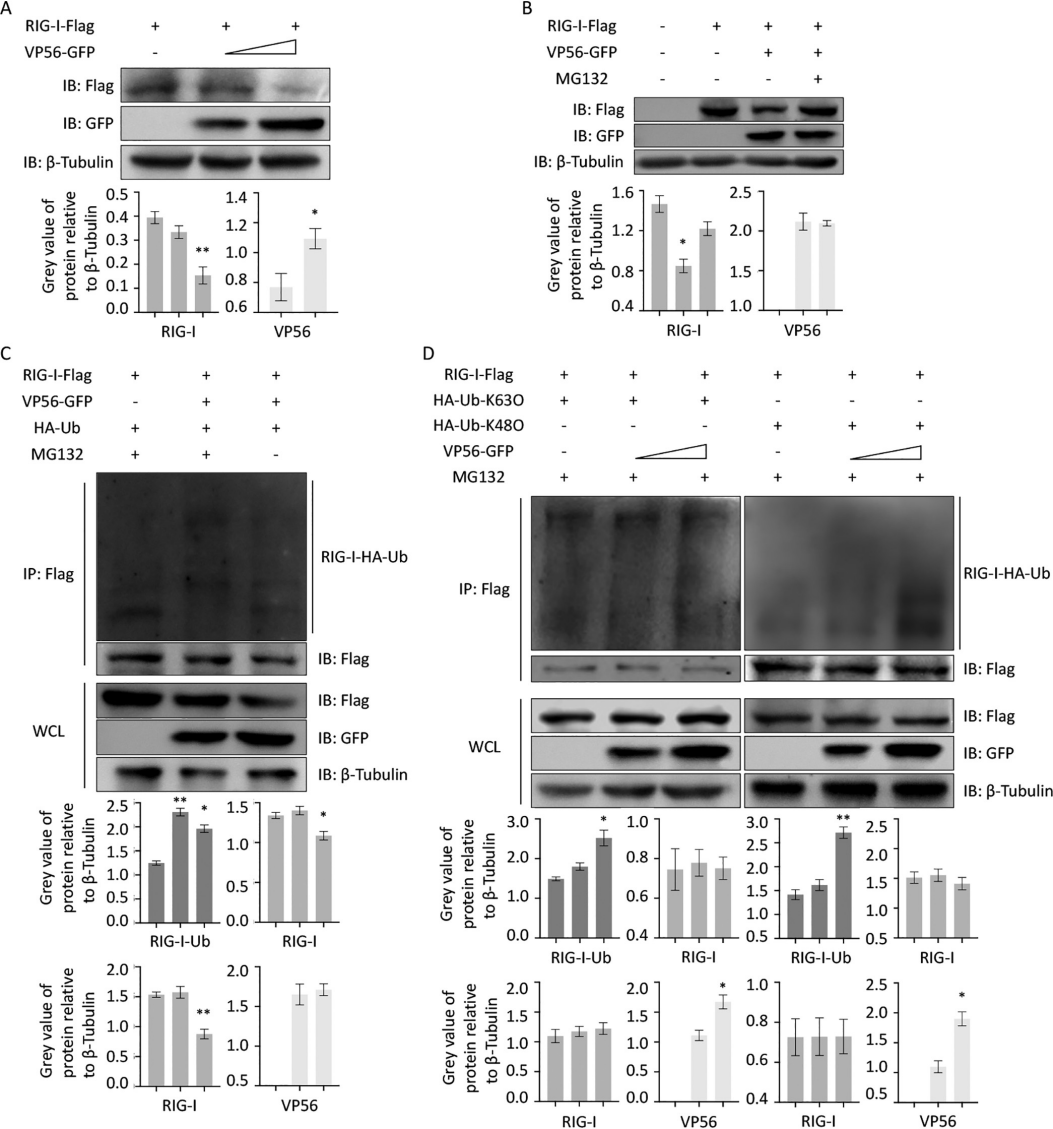
VP56 promotes RIG-I degradation via K48-linked ubiquitination. (A) VP56 degrades RIG-I protein. FHM cells were seeded into 6-well plates overnight and transfected with pRIG-I-Flag (1 μg each), pVP5-GFP (0, 1, 2 μg), and empty vector (2, 1, 0 μg). After 24 h, immunoblotting was performed with lysates and indicated Abs. (B) MG132 rescues VP56-induced RIG-I degradation. FHM cells were seeded into 6-well plates overnight and transfected with 1 μg of RIG-I-Flag, VP56-GFP, or empty vector. Eighteen hours posttransfection, dimethyl sulfoxide (DMSO) or MG132 (25 μM) was added for 6 h. Cells were harvested for immunoblotting with the indicated Abs. (C) VP56 promotes RIG-I ubiquitination. FHM cells were seeded in 10-cm^2^ dishes for 24 h and transfected with 2 μg of HA-Ub, VP56-GFP (0, 1.5, and 3 μg) together with empty vector (3, 1.5, and 0 μg), and 4 μg of RIG-I-Flag. At 42 h posttransfection, the cells were treated with MG132 for 6 h. The cells were then harvested for IP with Flag Ab and immunoblotting with the indicated Abs. (D) VP56 enhances K48-linked ubiquitination of RIG-I. FHM cells were seeded in 10-cm^2^ dishes for 24 h and transfected with 1 μg of HA-Ub-K63O or HA-Ub-K48O, VP56 (0, 1.5, and 3 μg) together with empty vector (3, 1.5, and 0 μg), and 4 μg of RIG-I-Flag. At 42 h posttransfection, the cells were treated with MG132 for 6 h. The cells were then harvested for IP with Flag Ab and immunoblotting with the indicated Abs. All experiments were repeated at least three times. The histograms below the immunoblotting results show the relative expression levels, which were quantified using ImageJ software.

Ubiquitination modifications of RIG-I play a pivotal role in the activation and attenuation of innate antiviral immunity. The effects of VP56 on expression and ubiquitination of RIG-I protein were investigated (Fig. 3). Protein expression of RIG-I decreased along with increasing VP56 (Fig. 3A), indicating that VP56 degrades RIG-I. MG132, a proteasomal pathway inhibitor, was then used to test whether VP56 degrades RIG-I via the proteasome pathway. The results demonstrated that MG132 rescues the inhibited RIG-I production by VP56, suggesting that VP56 degrades RIG-I through the proteasome pathway (Fig. 3B). Furthermore, ubiquitination assays were performed in FHM cells (Fig. 3C and D). These results showed that VP56 enhances ubiquitination of RIG-I (Fig. 3C). When K63- and K48-linked ubiquitination were measured, respectively, the results showed that VP56 enhances both K63- and K48-linked ubiquitination of RIG-I in a dose-dependent manner (Fig. 3D). Collectively, VP56 degrades RIG-I via K48-linked ubiquitination for the proteasomal pathway, and the immune-activated functions of K63-linked ubiquitination increased due to VP56 might be counteracted by RIG-I protein degradation because of VP56.

### VP56 inhibits RIG-I-triggered IFNs and inflammation responses.

**FIG 4:**
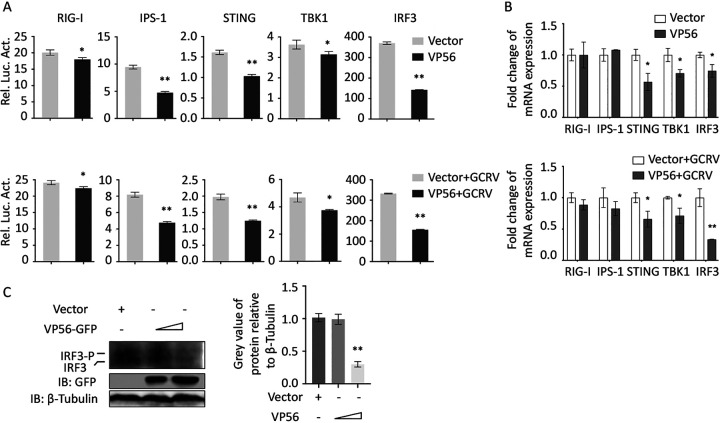
VP56 inhibits RLR signaling pathway key gene expression. (A) VP56 inhibits RLR signaling pathway key gene promoter activities. CIK cells seeded in 24-well plates overnight were cotransfected with 380 ng of VP56/empty vector, 380 ng of each target plasmid (pRIG-Ipro-Luc, pIPS-1pro-Luc, pSTINGpro-Luc, pTBK1pro-Luc, and pIRF3pro-Luc), and 38 ng of pRL-TK. Twenty-four hours later, the cells were uninfected (top) or infected with GCRV (bottom). The luciferase activities were examined at 24 h postchallenge. Rel. Luc. Act., relative luciferase activity. (B) VP56 decreases RLR-related gene mRNA expression in uninfected (top) or GCRV-infected (bottom) CIK cells. CIK cells transiently transfected with VP56/empty vector were seeded in 12-well plates. After 24 h, CIK cells were uninfected or infected with GCRV. Twenty-four hours postinfection, total RNAs were extracted, and mRNA expression was examined for RIG-I, IPS-1, STING, TBK1, and IRF3 genes. Data of reporter assays and qRT-PCR are shown as mean ± standard deviation (SD) of 4 wells of cells per group with three independent experiments. Significance was calculated in relation to the control group; *, *P* < 0.05; **, *P* < 0.01. The relative transcription levels were normalized to mRNA expression of the EF1α gene and are represented as fold change relative to the transcription level in control cells, which was set to 1. (C) VP56 suppresses IRF3 protein and phosphorylation levels. CIK cells transiently transfected with VP56/empty vector were seeded in 6-well plates. After 24 h, cell lysate was used for Western blotting using IRF3 polyclonal antiserum. β-Tubulin was used to normalize the protein concentration. All the experiments were repeated independently at least three times. The histograms beside the Western blotting results show the IRF3 and phospho-IRF3 (IRF3-P) expression levels, which were quantified using ImageJ software.

**FIG 5:**
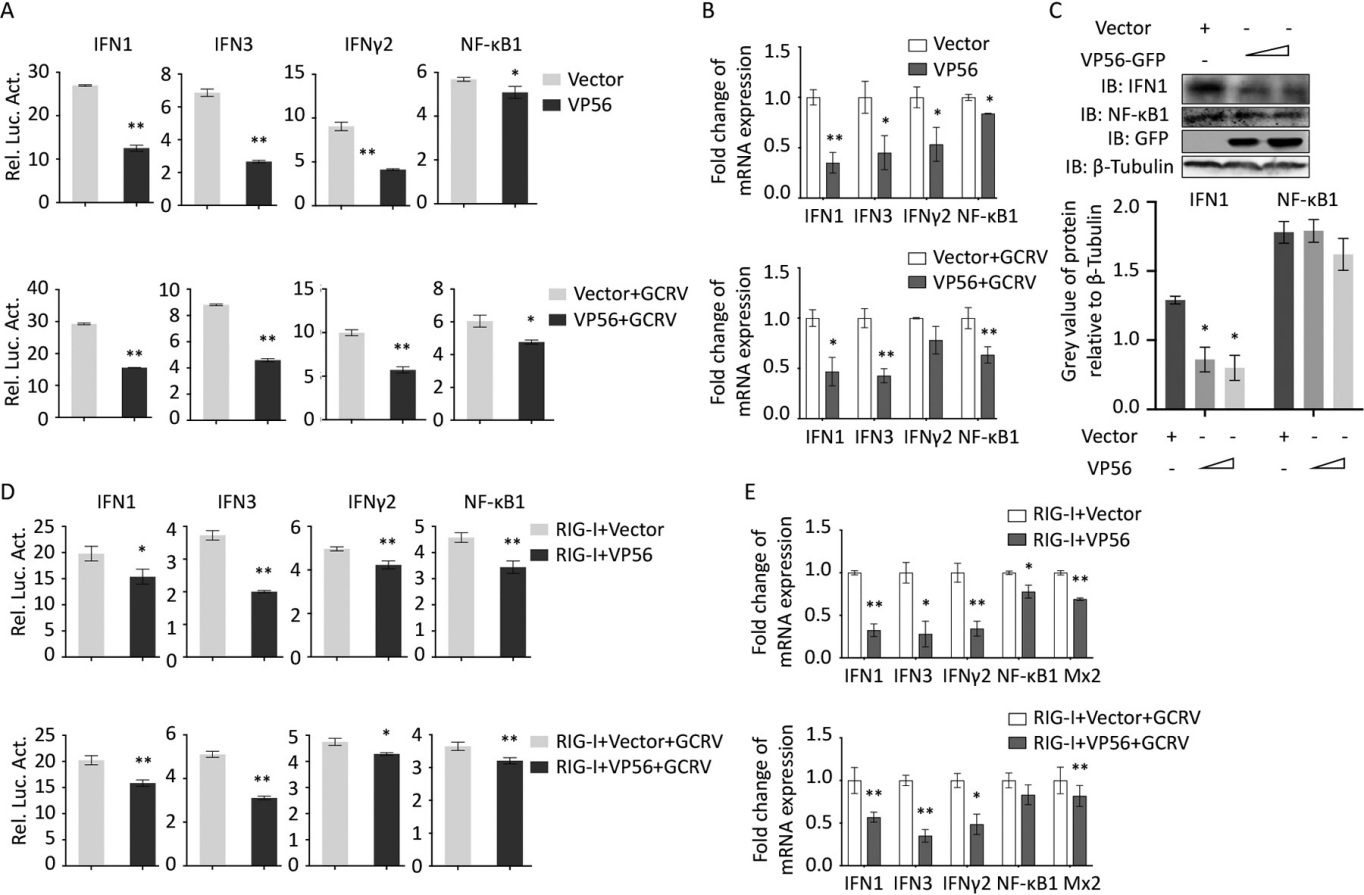
VP56 suppresses IFNs and inflammatory responses. (A) VP56 decreases IFN1, IFN3, IFN-γ2, and NF-κB1 promoter activities in uninfected (top) or GCRV-infected (bottom) CIK cells. (B) VP56 decreases IFN1, IFN3, IFN-γ2, and NF-κB1 mRNA expression levels in uninfected (top) or GCRV-infected (bottom) CIK cells. (C) VP56 reduces IFN1 and NF-κB1 protein expression. CIK cells transiently transfected with VP56/empty vector were seeded in 6-well plates. After 24 h, cell lysate was used for Western blotting using IFN1 or NF-κB1 polyclonal antiserum. The histogram exhibits the relative protein expression levels, which were quantified using ImageJ software. (D) VP56 inhibits IFNs and NF-κB1 promoter activities induced by RIG-I in uninfected (top) or GCRV-infected (bottom) CIK cells. (E) VP56 inhibits IFNs, NF-κB1, and antiviral effector Mx2 mRNA expression levels induced by RIG-I in uninfected (top) or GCRV-infected (bottom) CIK cells. All the experiments were repeated at least three times. Other figure captions are the same as Fig. 4A and B.

RIG-I is a crucial PRR in the antiviral signaling pathway, sensing viral dsRNA in the cytoplasm. VP56 binding and degrading of RIG-I is supposed to be beneficial for GCRV evasion and replication. At the promoter level, VP56 suppresses the promoter activities of the key molecules in the RLR signaling pathway, including RIG-I, IPS-1, STING, TBK1, and IRF3 by dual-luciferase assays (Fig. 4A). At the mRNA level, a similar tendency was observed by quantitative real-time RT-PCR (qRT-PCR) (Fig. 4B). Furthermore, IRF3 protein and phosphorylation levels were examined by Western blotting (WB). The results indicated that VP56 attenuates the protein expression and phosphorylation of IRF3 (Fig. 4C), which implied that the transcription of antiviral immune molecules would be reduced. Therefore, we further investigated the influence of VP56 on representative IFNs and inflammation factors at the promoter and transcription levels. The results indicated that VP56 reduces the promoter activities and mRNA expression of IFN1, IFN3, IFN-γ2, and NF-κB1 (Fig. 5A and B). Moreover, VP56 weakens the protein expression of IFN1 and NF-κB1 (Fig. 5C). In addition, when VP56 was cotransfected with RIG-I, the promoter activities and mRNA levels of IFN1, IFN3, IFN-γ2, and NF-κB1 were suppressed by VP56 compared with RIG-I alone (Fig. 5D and E), indicating that VP56 inhibits the RIG-I-triggered IFN and inflammatory responses.

### VP56 suppresses antiviral effectors and boosts GCRV evasion.

**FIG 6:**
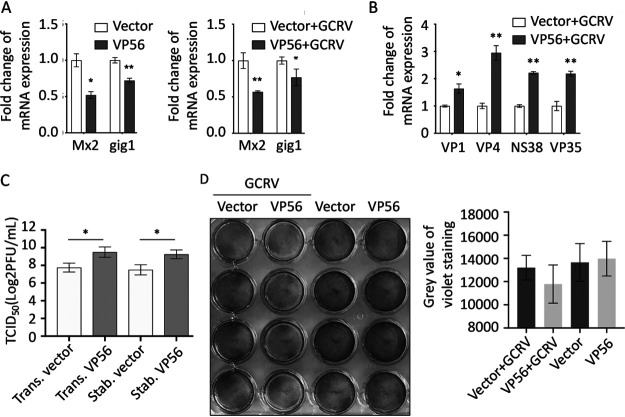
VP56 represses antiviral effectors and facilitates GCRV replication. (A) VP56 reduces antiviral effector mRNA expression in uninfected (left) or GCRV-infected (right) CIK cells. (B) VP56 facilitates mRNA expression of viral segments. Other figure captions are the same as Fig. 4B (C) VP56 promotes GCRV infection. CIK cells transiently transfected with empty vector/VP56 (trans. vector/trans. VP56) as well as stably transfected with empty vector/VP56 (stab. vector/stab. VP56) were seeded in 6-well plates overnight and infected with GCRV, and the supernatants were collected at 24 h postinfection for viral titer assays by TCID_50_. (D) VP56 facilitates GCRV-induced cell death. CIK cells transiently transfected with empty vector/VP56 were seeded in 24-well plates for 24 h, treated with PBS or infected with GCRV for 24 h, and fixed and stained with crystal violet. All the experiments were repeated at least three times. The histogram exhibits the relative crystal violet staining levels, which were quantified using ImageJ software.

To determine whether VP56 interferes with antiviral effectors downstream of the IFN pathway to facilitate viral evasion, we examined mRNA expression of representative IFN-stimulated genes (ISGs) (myxovirus-resistant 2 [*Mx2*] and GCRV-induced gene 1 [*gig1*]), viral genes (VP1, VP4, NS38, and VP35), viral titers (50% tissue culture infective dose [TCID_50_]), and cell death caused by VP56 (Fig. 6). mRNA expression of antiviral effectors *Mx2* and *gig1* were reduced by VP56 (Fig. 6A). After GCRV infection, viral VP1, VP4, NS38, and VP35, which are encoded by GCRV-II segment S1, S6, S10, and S11, respectively, were increased by VP56 (Fig. 6B). Furthermore, a TCID_50_ assay indicated that VP56 increases the titer of GCRV in CIK cells when transiently or stably transfecting VP56 (Fig. 6C). In addition, crystal violet staining also illustrated that VP56 promotes cell death caused by GCRV infection (Fig. 6D). All the results demonstrated that VP56 inhibits ISG expression and facilitates viral RNA synthesis, leading to increased GCRV titer and cytopathic effect.

### Confirmation of VP56-dependent repression of the RIG-I-triggered antiviral immune pathway.

**FIG 7:**
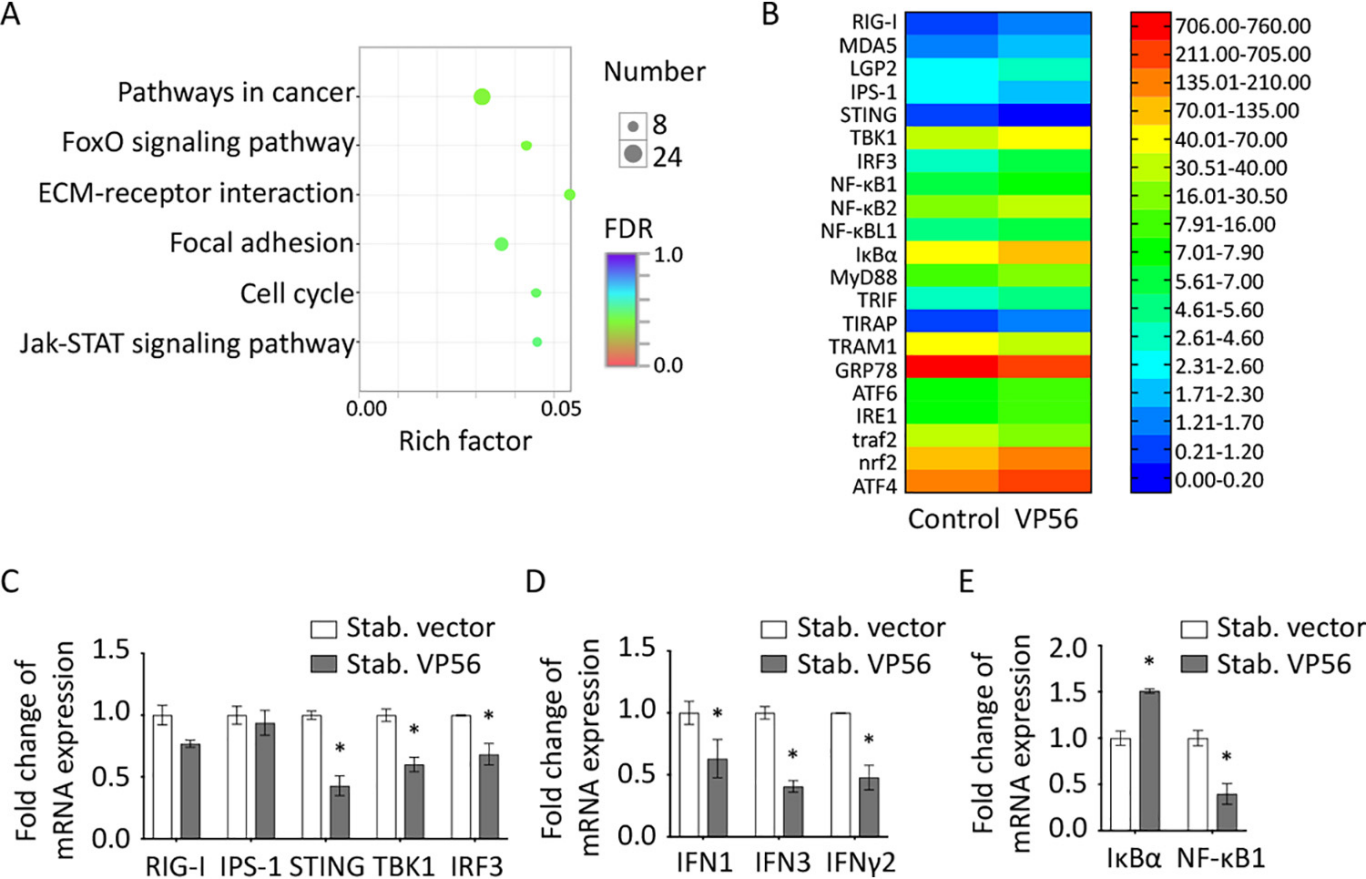
RNA-seq analyses and qRT-PCR verification with CIK cells stably expressing VP56. (A) Bubble chart of functional annotation of 4-fold differentially expressed genes based on KEGG categorization. The *y* axis indicates the signaling pathway category, and the *x* axis indicates the enrichment factor. (B) Heat map of raw gene expression of specific genes in the transcriptome database. Values on the right indicate FPKM (fragments per kilobase million). (C to E) Verification of gene expression. Transcriptome sample CIK cells that were stably expressed with empty vector (stab. vector) or VP56 (stab. VP56) were seeded into 12-well plates for 24 h and examined for the expression of RLR-related genes (C), IFNs (D), and NF-κB-related genes (E). Other figure captions are the same as Fig. 4B.

To further address the regulation mechanism of VP56 on the host antiviral signaling pathway, RNA sequencing (RNA-seq) was performed with VP56/empty vector stable expression in CIK cells (Fig. 7). KEGG functional enrichment of differentially expressed (fold change of at least 4) genes (DEGs) analysis showed that pathways in cancer, FoxO signaling, and extracellular matrix (ECM)-receptor interaction were activated the most (Fig. 7A), showing that these three signaling pathways have the strongest responses after VP56 stimulation. Raw expression levels of the RIG-I-related signaling pathway are shown in Fig. 7B, and representative genes (RIG-I, IPS-1, STING, TBK1, IRF3, IFN1, IFN3, IFN-γ2, NF-κB1, and IκBα) were selected for validation by qRT-PCR. The results showed that the RLR antiviral signaling pathway and inflammation factor NF-κB were inhibited by VP56 (Fig. 7C to E); meanwhile, expression of IκBα, an NF-κB suppressor gene, was enhanced (Fig. 7E).

**FIG 8:**
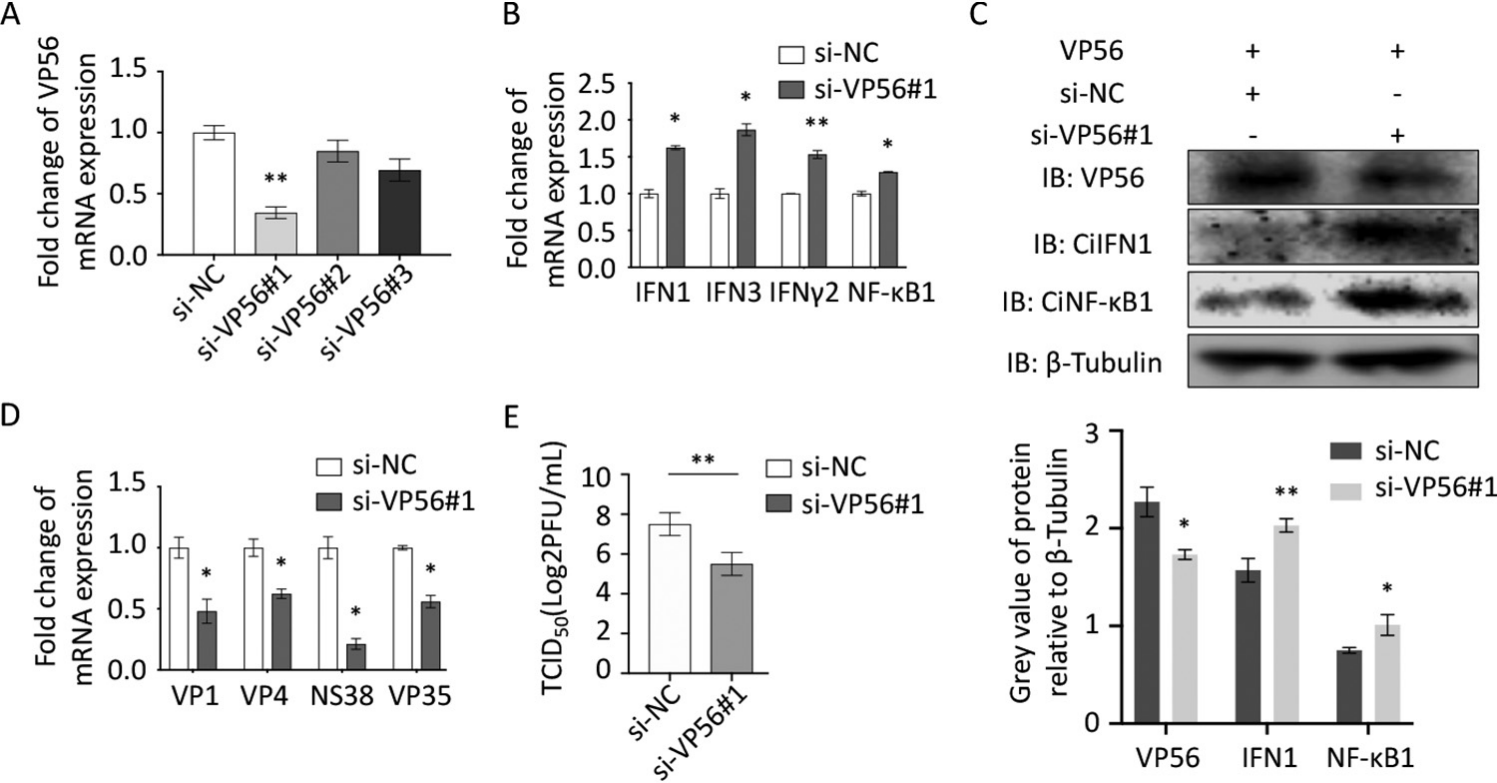
siRNA-mediated knockdown of VP56 in CIK cells stably expressing VP56 enhances antiviral immunity and restrains viral infection. (A) Screening the highly efficient siRNA by qRT-PCR. CIK cells stably expressing VP56 were seeded into 6-well plates overnight and transfected with 50 nM si-NC, si-VP56-1, si-VP56-2, or si-VP56-3 for 24 h, respectively. The total RNAs were extracted to examine mRNA expression of VP56. The experiment was repeated at least three times. (B to E) Effects of siRNA on mRNA expression of IFNs and NF-κB1 (B), protein levels of IFNs and NF-κB1 (C), the viral segment transcripts of GCRV (D), and viral titer (E). (B) CIK cells were seeded into 6-well plates overnight and transfected with 50 nM si-NC/si-VP56-1 for 24 h and infected with GCRV for 24 h. Total RNAs were isolated for qRT-PCR. (C) CIK cells were seeded into 6-well plates overnight and transfected with 50 nM si-NC/si-VP56-1 for 24 h. The cell lysates were collected for immunoblotting with IFN1 and NF-κB1 polyclonal antiserum. β-Tubulin was used as a control. The histogram shows the relative protein expression levels, which were quantified using ImageJ software. (D, E) VP56 knockdown reduces GCRV infection. CIK cells stably expressing VP56 were seeded in 6-well plates overnight, transiently transfected with si-NC or si-VP56-1 for 24h, and infected with GCRV for 24 h. The cells were collected for qRT-PCR assays of viral fragments (D), and the supernatants were gathered for viral titer assays by TCID_50_ (E). Other figure captions are the same as Fig. 4B.

VP56 knockdown potentiates IFN responses and reduces GCRV replication and titer (Fig. 8). The CIK cells that were stably transfected with VP56 were used to investigate the influence of VP56 knockdown on host antiviral immune genes and viral replication. si-VP56-1 displayed the highest interference efficiency among the three small interfering RNAs (siRNAs) by qRT-PCR (Fig. 8A). VP56 knockdown increases the representative IFN (IFN1, IFN3, and IFN-γ2) and inflammation factor (NF-κB1) responses (Fig. 8B). These results were further confirmed at the protein level (Fig. 8C). Furthermore, VP56 knockdown inhibits GCRV replication and titer (Fig. 8D and E). All the results indicated that VP56 knockdown enhances antiviral immunity and inhibits GCRV replication.

### VP56 allies VP4, synergistically facilitating viral evasion.

**FIG 9:**
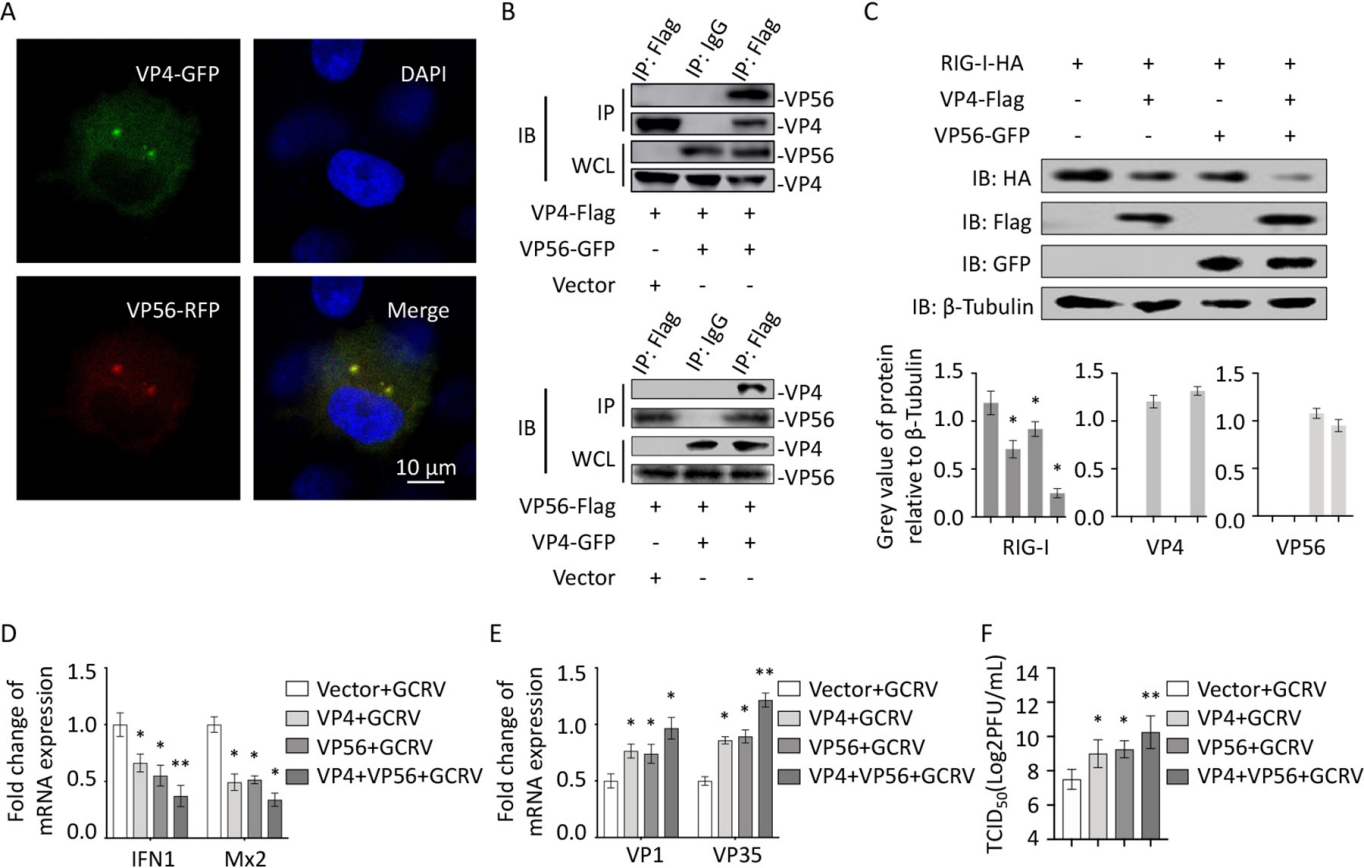
VP56 and VP4 bind together and more efficiently boost viral evasion. (A) VP56 colocalizes with VP4. FHM cells were cotransfected with VP4-GFP and VP56-RFP for 48 h, fixed, stained, and observed under a confocal microscope. (B) Co-IP between VP56 and VP4. Top, FHM cells were cotransfected with VP4-Flag and VP56-GFP/vector for 48 h. Co-IP was performed with Flag monoclonal Ab and mouse IgG (control) and immunoblotting with the corresponding Abs. Bottom, FHM cells were cotransfected with VP56-Flag and VP4-GFP/vector for 48 h. Co-IP was performed with Flag monoclonal Ab and mouse IgG (control) and immunoblotting with the corresponding Abs. (C) VP56 allies VP4 to more efficiently degrade RIG-I. FHM cells were cotransfected with RIG-I-HA, VP4-Flag, and VP56-GFP as indicated for 24 h, and immunoblotting was performed with lysates and indicated Abs. (D) VP56 and VP4 synergistically inhibit antiviral immunity. FHM cells were cotransfected as in Fig. 9C, and the total RNAs were prepared for qRT-PCR assays of IFN1 and Mx2 genes. (E) VP56 unites VP4 to more efficiently facilitate viral replication. FHM cells were cotransfected, and gene expression (VP1 and VP35) was quantified as in Fig. 9D. (F) VP56 and VP4 promote GCRV infection. CIK cells transfected with empty vector/VP4/VP56/VP4+VP56 were seeded in 6-well plates overnight and infected with GCRV, and the supernatants were collected at 24 h postinfection for viral titer assays by TCID_50_. All the experiments were performed in triplicate.

In our previous study, the GCRV major outer capsid protein VP4 binds to the CARD and RD domains of RIG-I, degrades RIG-I by the proteasomal pathway, and promotes viral replication (29). We therefore wondered whether there is some interaction between VP56 and VP4. We investigated the interaction and joint effect between VP56 and VP4 (Fig. 9). When VP4-GFP and VP56-red fluorescent protein (RFP) were cotransfected in FHM cells, we found that VP56 colocalizes with VP4 (Fig. 9A). Furthermore, co-IPs with VP56 pulling down VP4 and VP4 pulling down VP56 via different fusion tag Abs were performed, and the results demonstrated that VP56 and V4 bind together in the cytosol (Fig. 9B). After that, we cotransfected VP56 and VP4 and found that VP56 and VP4 synergistically degrade RIG-I (Fig. 9C), and mRNA expression of representative antiviral immune genes *IFN1* and *Mx2* were inhibited strongly (Fig. 9D); meanwhile, the expression of representative viral genes VP1 and VP35 was increased intensively (Fig. 9E). Titers of GCRV were increased by VP56 and VP4 synergistically (Fig. 9F). The results indicated that surface fibrin VP56 and major outer capsid protein VP4 of GCRV bind together and synergistically degrade RIG-I, suppress antiviral immune responses, and accomplish GCRV immune evasion.

## DISCUSSION

VP56 encoded by segment 7 is a fiber protein on the outer capsid of the GCRV-II/III particle (9), which is involved in cell attachment (10). VP56 is similar to the σ1 protein of mammalian reovirus (MRV) (8, 30). In mammals, junctional adhesion molecule A (JAM-A) is the only known cell surface receptor for MRV (31), but the function of JAM-A in fish is not certain (32). Several attempts have been made to find out the membrane receptor of aquareovirus, including fibulin-4, Ubc9, LITAF, LamR, TIA1, etc. (33–37). However, the receptor of GCRV-II on the cell surface is still not determined. Further research investigating GCRV fibrin may provide insight into GCRV infection mechanisms.

RIG-I is a crucial PRR recognizing viral RNA in the cytoplasm. Interaction between VP56 and RIG-I was found by endogenous co-IP, GST pulldown, and subsequent LC-MS/MS (Fig. 1B). All methods detected the binding. Subsequently, we further verified the interaction through traditional forward and reverse co-IPs (Fig. 2A) and a far-red mNeptune-based bimolecular fluorescence complementation system (Fig. 2B), which is a novel visible technique to research molecular interaction (38). The helicase domain of RIG-I is critical for recognition of RNA, binding to the sugar-phosphate backbone of duplexed RNA, which results in release of CARDs (39). The location of the binding site in the helicase domain (Fig. 2C) implies that VP56 may interfere with RIG-I binding dsRNA, which may affect the RIG-I-mediated antiviral signaling pathway.

It is widely believed that many host cellular physiologic processes are regulated by the ubiquitin system. It is of great importance in protein stability, immune activation, and host-pathogen interactions (12). In mammals, RIG-I posttranslational modification by K63-linked or K48-linked ubiquitination is important for immune regulation functions (40). In fishes, K63- or K48-linked RIG-I ubiquitination shows similar functions in mammals (22, 29, 41). Tripartite motif containing 25 (TRIM25) and Riplet mediate K63-linked ubiquitination in residues in RIG-I-CARDs for downstream IPS-1 recruitment and signal transduction (42). The K48-conjugated ubiquitination chain is mediated by RNF125 to deliver substrates to proteasomes for degradation. This process guarantees a basal protein level for subsequent rapid signal activation (43). GCRV-II VP56 induces both K63- and K48-linked ubiquitination in the present assays (Fig. 3). The K48-linked proteasomal degradation pathway induced by VP56 plays a stronger role in the effect on RIG-I, leading to reduced downstream IFNs and inflammatory responses (Fig. 4 and 5). These results demonstrate that VP56 aims at and degrades RIG-I to weaken downstream signaling transduction via posttranslation modification.

RIG-I senses viral RNA, which triggers downstream cascade signals through IPS-1, STING, TBK1, and IRFs to promote IFN production for antiviral responses (44). Independent of GCRV infection, VP56 inhibits RIG-I and downstream signaling molecules at promoter and mRNA levels (Fig. 4A and B). Downstream IRF3 was obviously suppressed by VP56 at protein and phosphorylation levels in a dose-dependent manner (Fig. 4C). Correspondingly, IFNs were reduced by VP56 at mRNA and protein levels (Fig. 5). As a result, antiviral effectors were repressed, and GCRV accomplished immune evasion (Fig. 6). Additionally, when cotransfected with RIG-I, VP56 blocks RIG-I-triggered IFNs at promoter and mRNA levels, suggesting that VP56 targets RIG-I (Fig. 5D and E). VP56 also reduces NF-κB expression (Fig. 5), whose translocation from the cytoplasm to the nucleus induces the expression of proinflammatory cytokines. These results were further confirmed by RNA-seq (Fig. 7) and siRNA experiments (Fig. 8). The present study revealed that fibrin VP56 targets and degrades the RNA sensor RIG-I, thereby attenuating the signaling pathway and antiviral effectors, boosting viral immune evasion.

The IFN response is of great significance in host innate immunity against both viral and bacterial infections (45). The RLR-mediated IFN signaling pathway is an important target for viral antagonism or escape to facilitate viral infection (46, 47). Herpes simplex virus 1 (HSV-1) tegument protein UL37 targets the helicase domain of RIG-I to deamidate RIG-I, inhibiting RNA-induced activation of innate immune signaling (28). STING is an important molecule in the RLR-IFN pathway and is often targeted by viruses. Hepatitis C virus (HCV) NS4B, a nonstructural protein, interacts with STING protein to restrain IFN responses (48). NS2B3, a protease of dengue virus (DENV), inhibits IFN production via cutting STING (49, 50). Papain-like proteases (PLP) of human coronavirus (HCoV) NL63 and severe acute respiratory syndrome coronavirus (SARS-CoV) inhibit IRF3 activation by destroying the STING dimer, thereby antagonizing innate immune signal transduction (51). TBK1 is another important target for virus antagonism in the RLR-IFN pathway (52). PLP2 of mouse hepatitis virus A59 (MHV-A59) affects polyubiquitination modification of TBK1 to block its kinase activity (53). NS3 and NS2 proteins of HCV physically interact with TBK1 to competitively inhibit activation of downstream IRF3 (54). IRF3/7 are crucial IFN regulation factors. In a previous report, GCRV VP56 represses IFN production by degrading phosphorylated IRF7 (11). In the present study, we found that VP56 targets and degrades the immune initial point molecule RIG-I (Fig. 3A) and boosts viral evasion (Fig. 6B); meanwhile, it also decreases protein and phosphorylation levels of IRF3 (Fig. 4C).

Major outer capsid protein VP4 of GCRV has been reported to localize at the early endosome, lysosome, and endoplasmic reticulum (ER) but not the late endosome, which is the same location as VP56 (29). VP4 also binds to RIG-I but at different domains (CARDs and RD) and promotes K48-linked ubiquitination of RIG-I to degrade it, resulting in low IFN responses (29). VP56 and VP4 bind together in the cytosol, localize at the early endosome, lysosome, and ER, recruit and wrap the three domains of RIG-I, degrade RIG-I by the proteasomal pathway, and unite to synergistically suppress antiviral immunity and more effectively facilitate viral replication; this demonstrates that two viral outer proteins cooperatively aim at the initial point molecule of immune responses, RIG-I, in the process of GCRV infection, synergistically inhibiting antiviral immunity and more efficiently accomplishing viral immune evasion.

**FIG 10:**
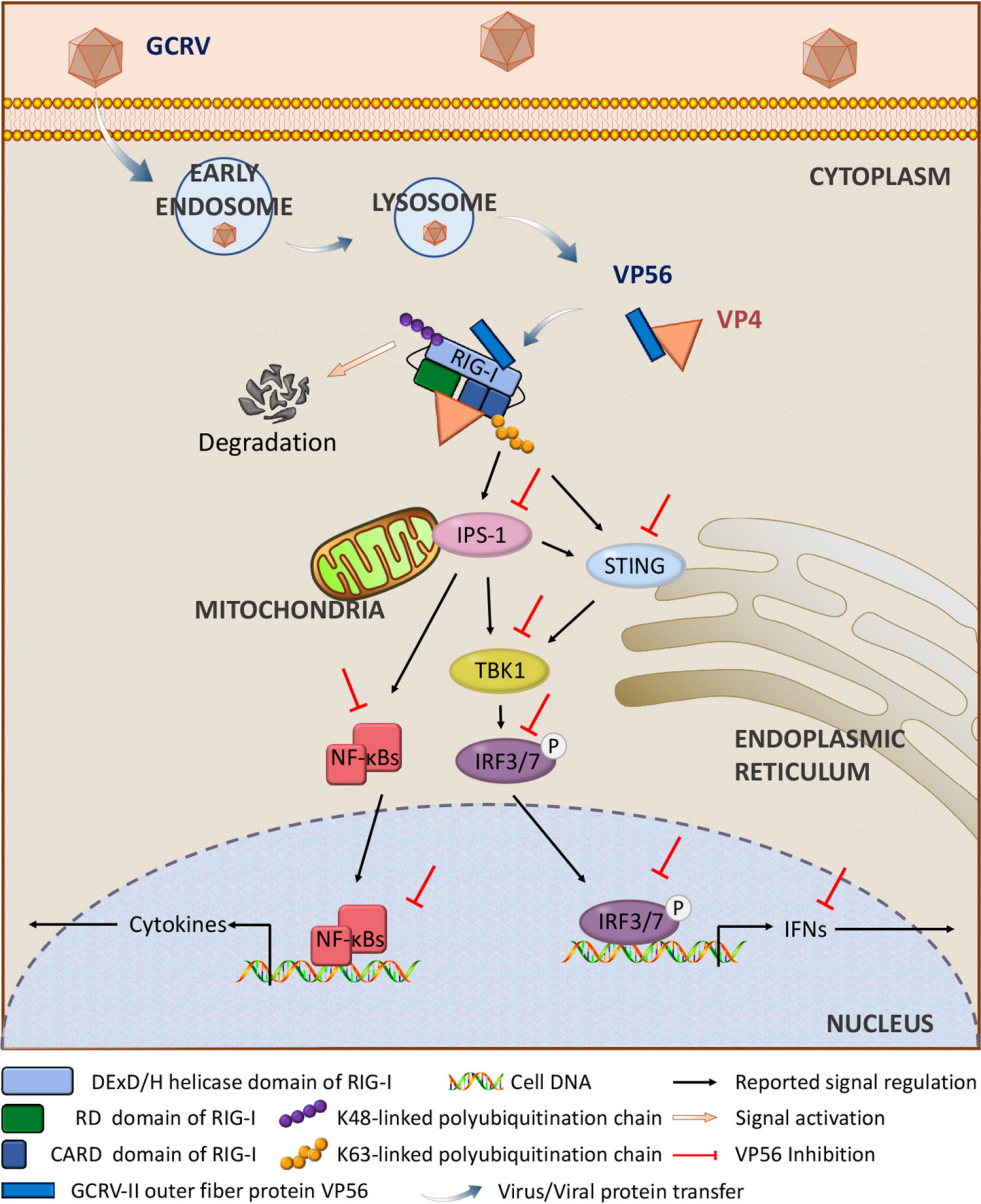
Illustration of VP56 in the regulation of the antiviral signaling pathway in grass carp. Following GCRV-II/GCRV-III infection, fibrin VP56 and major outer capsid protein VP4 bind together in the cytosol and disperse at the early endosome, lysosome, and ER, etc., where they recruit the viral RNA sensor RIG-I at different regions (VP56 binds to the helicase domain, VP4 binds to CARDs and RD [29]), form a shield, and obstruct RIG-I sensing of viral RNA. Meanwhile, they synergistically enhance the K48-linked ubiquitination of RIG-I to degrade RIG-I by the proteasomal pathway. Signal transduction from RIG-I to downstream adaptors IPS-1 and STING, TBK1, and IRF3 are then attenuated. Furthermore, VP56 restrains protein expression and phosphorylation of IRF3 and degrades IRF7 (11), thereby inhibiting the subsequent signals of IFNs and NF-κB. Consequently, antiviral effectors are suppressed, and GCRV accomplishes immune evasion and infection.

The present work reveals that GCRV fibrin VP56 acts as a forceful weapon for GCRV infection to inhibit IFN responses and boost viral evasion (Fig. 10). GCRV invades cells, the virus uncoats actively or passively, and outer fibrin VP56 is released and disperses at the early endosome, lysosome, ER, etc., where VP56 and VP4 bind together, recruit and wrap the different regions (helicase domain for VP56, CARDs and RD for VP4) of the viral RNA sensor RIG-I (one of the initial point molecules of antiviral immunity), block and prevent RIG-I from sensing viral RNA, and, furthermore, facilitate K48-linked ubiquitination of RIG-I to degrade it via the proteasomal pathway, thus the initial immune function of RIG-I is impeded by dual mechanisms (blockade and degradation). The inhibited RIG-I signal leads to restrained IFN antiviral responses and enhanced GCRV replication and infection. VP56 cooperates with VP4 to synergistically reduce host antiviral immunity during GCRV infection to more effectively accomplish viral immune evasion. These results clarify the functions and mechanisms of VP56 in GCRV infection and reveal an escaping strategy of RNA virus, providing insight into the mechanisms of viral evasion and antiviral immunity.

## MATERIALS AND METHODS

### Cells.

CIK (*C. idella* kidney) and FHM (fathead minnow) cells were respectively cultured in Dulbecco’s modified Eagle’s medium (DMEM) and M199 supplemented with 10% fetal bovine serum (FBS) (Gibco), 100 U/ml penicillin (Sigma), and 100 μg/ml of streptomycin (Sigma). Cells were incubated at 28°C with 5% CO_2_ humidified atmosphere. Stably expressed CIK cell lines were screened with G418.

### Virus and infection.

The GCRV-II GCRV-097 strain was conserved in our lab. For viral infection, CIK cells were plated for 24 h in advance and then infected with GCRV-097 at a multiplicity of infection (MOI) of 1 as previously described (41).

### TCID_50_ assay.

Samples were infected with the GCRV-097 strain (MOI = 1) for 24 h. The supernatants were serially diluted 10-fold and incubated with CIK cells in a flat 96-well plate to determine the 50% tissue culture infective dose (TCID_50_). Cells were incubated at 28°C for 7 days. On day 7, the plates were examined for the presence of viral cytopathic effect under the microscope.

### Antibodies.

Mouse IFN1 polyclonal antibody (Ab) was prepared and conserved by our lab. Anti-IRF3 rabbit polyclonal antiserum was previously prepared and presented by Yibing Zhang, Institute of Hydrobiology, Chinese Academy of Sciences, Wuhan, China (25). These Abs were tested by Western blotting before experiments. The VP56 Ab was produced and conserved in our lab. Hemagglutinin (HA) tag (ab18181) mouse monoclonal Ab, Flag tag (ab125243) mouse monoclonal Ab, and β-tubulin rabbit polyclonal Ab (ab6046) were purchased from Abcam. GFP mouse monoclonal Ab (AE012) and GST mouse monoclonal Ab (AE001) were purchased from Abclonal. IRDye 800CW donkey anti-rabbit-IgG (926-32213) and anti-mouse-IgG(H+L) (926-32212) secondary Abs were purchased from LI-COR. Goat anti-mouse IgG horseradish peroxidase (HRP)-conjugated secondary Ab (A0216) was purchased from Beyotime.

### Plasmid construction.

The plasmids pGEX-4T-1 and pCMV-GFP were used for construction of expression vectors with specific primers (Table S2 in the supplemental material). VP56/VP56-Flag was ligated into pGEX-4T-1 and pCMV-GFP, respectively, to construct expression vectors. Expression plasmids RIG-I-HA, RIG-I-Flag, RIG-I-CARD-HA, RIG-I-helicase-HA, and RIG-I-RD-HA were saved in our lab (41). For dual-luciferase reporter assays, the valid promoters (RIG-I, IPS-1, STING, TBK1, IRF3, IFN1, IFN3, IFN-γ2, and NF-κB1, respectively) were cloned into pGL3 basic luciferase reporter vector (Promega), which had been previously constructed in our lab (41, 55). HA-Ub, HA-Ub-K63O, and HA-Ub-K48O plasmids were kindly provided by Hong-Bing Shu (Wuhan University, Wuhan, China).

### Recombinant expression and purification of VP56.

Full-length GCRV-097 VP56 was amplified with corresponding primers (Table S2) and cloned into pGEX-4T-1 vector. The plasmid pGEX-4T1-VP56 was transformed into E. coli BL21(DE3) pLysS cells for prokaryotic expression. The fusion protein was induced by isopropyl-β-d-1-thiogalactopyranoside (IPTG) and purified by GST-bind resin (Genscript) chromatography.

### Endogenous coimmunoprecipitation, GST pulldown, and LC-MS/MS.

Co-IP assays and GST pulldown were performed to explore CIK cell proteins interacting with VP56. For co-IP, CIK cells were infected with GCRV for 24 h, and co-IP was performed with GST Ab, GST-VP56 Ab, or negative serum (NS) using a coimmunoprecipitation kit (Pierce). The eluant was analyzed by SDS-PAGE and subsequent silver staining.

For GST pulldown, CIK cell proteins were extracted with a membrane and cytosol protein extraction kit (Beyotime). Two hundred microliters of GST-VP56 and 200 μl of CIK protein solutions (1 μg/μl, diluted in Tris-buffered saline [TBS]) were incubated at 4°C for 30 min, and then GST-bind resin (20 μl) was added. After 6 h of incubation at 4°C, the resin was washed with TBS thoroughly and eluted with elution buffer (10 mM reduced glutathione and 50 mM Tris-HCl, pH 8.0) and then analyzed using SDS-PAGE and subsequent silver staining.

After co-IP or GST pulldown, LC-MS/MS analysis was respectively performed on a Q Exactive mass spectrometer (Thermo Scientific) by Shanghai Applied Protein Technology Co. Ltd. LC-MS/MS spectra were searched using the MASCOT engine (Matrix Science) against the actinopterygii UniProt sequence database (http://www.uniprot.org/), Grass Carp Genome Database (GCGD) (http://bioinfo.ihb.ac.cn/gcgd/php/index.php), and a grass carp transcriptome database in the NCBI SRA browser (BioProject accession number SRP049081) (56).

### Western blotting.

For Western blotting, protein extracts were separated by 8–12% SDS-PAGE gels and transferred onto nitrocellulose membranes (Millipore). The membranes were blocked in fresh 2% bovine serum albumin (BSA) dissolved in Tris-buffered saline with Tween 20 (TBST) buffer at 4°C overnight and incubated with appropriate indicated primary Abs for 2 h at room temperature. They were then washed three times with TBST buffer and incubated with secondary Ab for 1 h at room temperature. After washing four times with TBST buffer, the nitrocellulose membranes were scanned and imaged by an Odyssey CLx imaging system (LI-COR) or an ImageQuant (GE). The results were performed in triplicate.

### Traditional co-IP analysis.

For co-IP, CIK cells in 10-cm^2^ dishes were cotransfected with the indicated plasmids for 48 h. The cells were lysed in IP lysis buffer (20 mM Tris [pH 7.4], 150 mM NaCl, 1% Triton X-100, 1 mM EDTA, 1 mM Na_3_VO_4_, 0.5 mg/ml leupeptin, and 2.5 mM sodium pyrophosphate) (Beyotime) added with 1 mM phenylmethylsulfonyl fluoride (PMSF) for 30 min on ice, and the cellular debris was removed by centrifugation at 12,000 × *g* for 30 min at 4°C. The supernatant was transferred to a fresh tube and incubated with 1 μg of Ab with gentle shaking overnight at 4°C. Protein A+G Sepharose beads (Beyotime) (30 ml) were added to the mixture and incubated for 2 h at 4°C. After centrifugation at 3,000 × *g* for 5 min, the beads were collected and washed four times with lysis buffer. Subsequently, the beads were suspended in 20 ml of 2× SDS loading buffer and denatured at 95°C for 10 min followed by Western blotting.

### Ubiquitination assay.

For ubiquitination detection, FHM cells were seeded in dishes and transfected with corresponding plasmids. At 48 h posttransfection, the cells were treated with MG132 (Selleck) for another 6 h. The cells were then harvested for IP with Flag Ab and immunoblotted with HA and Flag Ab, respectively. The experiments were repeated at least three times. The histogram exhibits the relative protein expression levels, which were quantified using ImageJ software.

### Far-red mNeptune-based bimolecular fluorescence complementation system.

A far-red mNeptune-based bimolecular fluorescence complementation (BiFC) system is used to determine whether two proteins are interactive based on reconstitution of two nonfluorescent fragments of a fluorescent protein (38). In the present study, the mNeptune-based BiFC system was used to visualize the interaction between VP56 and RIG-I in CIK cells. Briefly, the open reading frame (ORF) sequences of VP56 and RIG-I were amplified and inserted into the pMN155 and pMC156 plasmids, respectively. The final plasmids were named pVP56-MN155 and pMC156-RIG-I, which contain the N-terminal domain of mNeptune (mNeptune amino acids 1 to 155; MN155) behind the C-terminal domain of VP56 and the C-terminal domain of mNeptune (mNeptune amino acids 156 to 244; MC156) in front of the N-terminal domain of RIG-I, respectively. The coding regions were connected by the linker sequence GGGGSGGGGS. Plasmids pVP56-MN155 and pMC156-RIG-I were then transfected into CIK cells alone or together as described above. mNeptune was observed by confocal microscopy. Red mNeptune BiFC signals were measured with excitation at 640/20 nm and emission at 685/40 nm.

### Dual-luciferase reporter assay.

CIK cells were seeded in 24-well plates for 24 h. Cotransfection was performed with corresponding expression plasmid, target promoter luciferase plasmid, and internal control reporter vector (pRL-TK). At 24 h posttransfection, cells were infected with GCRV or uninfected for 24 h. The cells were washed with phosphate-buffered saline (PBS) and lysed with passive lysis buffer (Promega) for 30 min. Luciferase activities were detected by a dual-luciferase reporter assay system (Promega). The luciferase reading was normalized against those in the pRL-TK levels, and the relative light unit intensity was presented as the ratio of luciferase of firefly to renilla.

### Transcriptome analyses and qRT-PCR verification.

Transcriptome data of empty vector stably transfected or VP56 stably transfected CIK cells were derived from previous studies performed by our lab and deposited in transcriptome databases (NCBI SRA number SRP212372, sample SAMN12168356/SAMN12168358) (29). Transcriptome data were verified for the effect of VP56 on the RLR signaling pathway and related IFN responses at the mRNA level. Gene expression levels according to the transcriptome data were validated by qRT-PCR.

### Crystal violet staining.

For crystal violet staining, CIK cells were seeded into 24-well plates overnight and transiently transfected with empty vector or VP56. At 24 h posttransfection, cells were infected with GCRV or uninfected. At 24 h postinfection, cells were washed and fixed with 4% paraformaldehyde for 15 min at room temperature and stained with 0.05% (wt/vol) crystal violet (Sigma, USA) for 30 min then washed with water and drained. Finally, the plates were photographed under a light box (Bio-Rad).

### siRNA-mediated knockdown of VP56.

CIK cells stably expressing VP56 were used for VP56 knockdown assays. Transient knockdown of VP56 was achieved by transfection of siRNA targeting VP56 mRNA. Three siRNA sequences (si-VP56-1 [sense 5′ to 3′], CUCCACAACUUUAGAUGAATT, si-VP56-2 [sense 5′ to 3′], CCUAUAGCCGUCGCUAAAUTT, and si-VP56-3 [sense 5′ to 3′], GGAGGAAGCAUUUGUAGGUTT) targeting different regions of VP56 were synthesized by GenePharma (Jiangsu, China). CIK cells were transfected with siRNA using GP-siRNA-Mate Plus (GenePharma, China) for 24 h. Silencing efficiencies of the candidate siRNAs were evaluated by qRT-PCR, and results were compared with those in the negative-control siRNA (si-NC) provided by the supplier. A preliminary experiment indicated that si-VP56-1 possessed the best silencing efficiency at a final concentration of 100 nM at the mRNA level. For Western blotting, CIK cells stably expressing VP56 were plated in 6-well plates and transfected with si-VP56-1 using GP-siRNA-Mate Plus (GenePharma, China) for 24 h and infected with GCRV for another 24 h. The cells were lysed, and proteins were extracted for Western blotting.

### Quantitative RT-PCR assay.

Total RNAs were prepared according to a previous report (57). Quantitative RT-PCR (qRT-PCR) was performed using a Roche LightCycler 480 system, and EF1α was used as an internal control gene for cDNA normalization. qRT-PCR amplification was performed in a total volume of 15 μl containing 7.5 μl of BioEasy master mix (SYBR green) (Hangzhou Bioer Technology Co., Ltd.), 3.1 μl of nuclease-free water, 4 μl of diluted cDNA (200 ng), and 0.2 μl of each gene-specific primer (10 μM) (Table S2).

### Statistical analysis.

The data were analyzed as previously described (58). Briefly, data were analyzed using an unpaired, two-tailed Student’s *t* test. *P* values below 0.05 were regarded as being significant for all analyses (*, *P* ≤ 0.05; **, *P* ≤ 0.01).
