# Supplementary material for: Grass Carp Reovirus VP56 Allies VP4, Recruits, Blocks, and Degrades RIG-I to More Effectively Attenuate IFN Responses and Facilitate Viral Evasion
Source: Microbiol Spectr. 2021 Sep 15;9(2):e01000-21. doi: 10.1128/Spectrum.01000-21 (PMC8557896; doi:10.1128/Spectrum.01000-21)
Supplement: SUPPLEMENTAL FILE 1 — Supplemental material. Download Spectrum.01000-21-s0001.pdf, PDF file, 1.2 MB [file spectrum.01000-21-s0001.pdf]

Supplemental Material FOR Publication

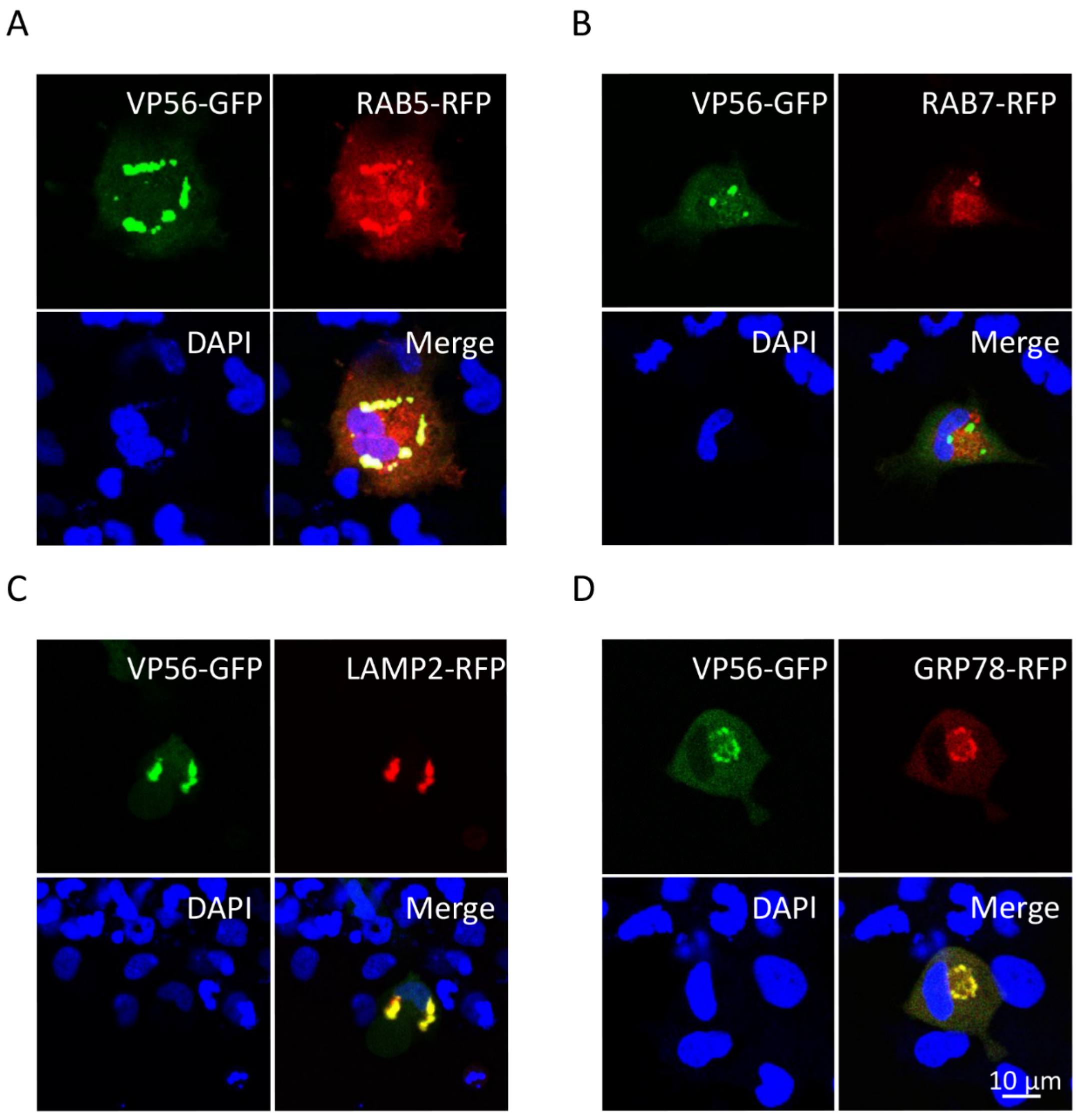

Figure S1. VP56 localizes at early endosome, lysosome and endoplasmic reticulum (ER), but not late endosome. FHM cells were respectively transiently co-transfected with VP56-GFP and LAMP2 (lysosome protein marker), VP56-GFP and RAB5-RFP (early endosome protein marker), VP56-GFP and RAB7-RFP (late endosome protein marker) or VP56-GFP and GRP78 (ER protein marker), then were seeded on observation dishes for confocal microscopy examination. After 48 h, the cells were

fixed with 4% (v/v) paraformaldehyde and stained with DAPI. All samples were subsequently visualized using a confocal microscope. Green signals represent VP56, and red signals stand for RAB5, RAB7, LAMP2, or GRP78. The blue staining indicates nucleus. The yellow signals in the merged images indicate the co-localization between VP56 and organelle (original magnification × 40). All the experiments were repeated independently at least three times.

Table S1

Proteins identified to potentially interact with VP56 according to co-IP/GST-pull down and subsequent LC-MS/MS analysis aligning with Uniprot-Spirulina, Uniprot-grass carp and amino acid database translated from grass carp genome and transcriptome as well as GCRV-HZ08 genome.

UniProtKB	Gene ID	Gene name	Number of peptides	Sequence coverage
	ADJ75338.1	VP56	59	42.15%
P18520		Intermediate filament protein ON3	9	10.38%
H2SIQ3		Uncharacterized protein	6	2.60%
W5N831		Uncharacterized protein	7	4.65%
A0A1A8N3C8		ATP synthase subunit alpha	5	9.26%
A0A1W4ZFF8		14-3-3 protein theta-like	3	11.84%
A0A2D0S8R3		E3 ubiquitin-protein ligase rnf213-beta-like	3	0.44%
I3JS45		Uncharacterized protein	3	4.11%
Q6PHE4		Zgc:77517	3	5.93%
A0A1A8E8B7		Protocadherin 2 alpha c (Fragment)	5	3.50%
A0A1A8V3H7		Uncharacterized protein	3	3.18%
H2V0L7		Uncharacterized protein (Fragment)	3	4.57%
I3J521		Uncharacterized protein	3	4.94%
Q4SL93		Chromosome 7 SCAF14557, whole genome shotgun sequence	3	4.15%
W5KXP1		Wu:fk65c09	3	1.87%
W5K5R3		Elongation factor 1-alpha	7	14.04%
Q5KQS8		Beta cytoplasmic actin	7	17.60%
A0A1S3R544		Glyceraldehyde-3-phosphate dehydrogenase	6	17.61%
H2MSJ5		Uncharacterized protein	5	14.25%
A0A1A8ECU4		Solute carrier family 25 (Mitochondrial carrier, adenine nucleotide translocator), member 6 (Fragment)	5	20.19%
A0A146NVI8		Heat shock cognate 71 kDa protein	4	6.79%
D5FQ07		Cytochrome c	4	32.69%
H2URZ5		Histone H4	4	15.88%
A0A2I4D0S5		cytochrome c isoform X1	3	18.11%
I3K289		Uncharacterized protein	3	15.23%
W5MDN9		Uncharacterized protein	3	1.89%
W5N831		Uncharacterized protein	3	3.55%
A0A1A7WW49		Histone H2B	3	12.90%
F8QPI3		Retinoic acid-inducible protein I	2	0.95%
M4QQY1		Retinoic acid inducible protein I	2	0.95%
P83751		Actin, cytoplasmic 1	6	15.73%
G3F1Q2		HSC70	3	4.16%
B7U616		GRP78	3	4.13%
P83751		Actin, cytoplasmic 1	13	34.93%

F8QMS8		Elongation factor 1-alpha (Fragment)	4	8.59%
F4YAX3		Heat shock 70 kDa protein	3	5.29%
B7U616		GRP78	3	5.21%
	CI01000021_03382666_03386768	Keratin 8	10	12.71%
	CI01000021_03428718_03432817	keratin 5 isoform X1	10	16.48%
	CI01000340_09015910_09020996	keratin, type II cytoskeletal 8-like isoform X1	10	10.10%
	CI01000300_00474917_00476427	actin, cytoplasmic 2	6	17.72%
	CI01000305_00342924_00346089	keratin, type I cytoskeletal 19-like	5	5.12%
	CI01000187_00277107_00282018	ATP synthase subunit alpha, mitochondrial-like	4	7.36%
	CI01000340_09053887_09057663	keratin, type I cytoskeletal 18-like	4	10.05%
	CI01000009_12863333_13023317	titin	3	0.05%
	CI01000010_11082426_11093899	keratin, type II cytoskeletal 8 isoform X1	3	3.27%
	CI01000001_05803416_05809184	sarcolemma associated protein b isoform X1	3	5.88%
	CI01000021_03324720_03328120	keratin, type I cytoskeletal 18	3	3.19%
	CI01000069_00000091_00001650	heat shock 70 kDa protein	3	5.19%
	CI01000046_03794654_03799486	Actin, cytoplasmic 2	11	27.52%
	CI01000021_02290326_02297117	elongation factor 1-alpha 2	7	12.31%
	CI01000051_05314994_05317995	cardiac muscle alpha actin 1	6	17.20%
	CI01000021_03382666_03386768	Keratin 8	6	5.31%
	CI01000000_11600258_11602519	ADP/ATP translocase 2	5	11.35%
	CI01000354_01178068_01179530	Histone H4	5	16.25%
	CI01000024_03042658_03044136	cytochrome c	4	25.00%
	CI01000054_04411682_04448848	nesprin-1 isoform X13	4	0.29%
	CI01000179_01205188	histone H3-like	4	15.68%

	_01208430			
	CI01000001_11019871_11028065	myosin light polypeptide 6-like isoform X1	3	8.91%
	CI01000069_00000091_00001650	heat shock 70 kDa protein	3	6.54%
	CI01000135_00679808_00684801	GTPase IMAP family member 8-like	3	2.29%
	comp61180_c0_seq1	keratin 5	11	13.13%
	comp56057_c0_seq1	keratin 8, partial	10	14.11%
	comp47208_c0_seq1	actin, cytoplasmic 2	6	15.73%
	comp53152_c0_seq1	PREDICTED: keratin, type I cytoskeletal 19-like	5	5.39%
	comp35118_c0_seq1	keratin, type I cytoskeletal 18-like	4	10.39%
	comp3163_c0_seq1	sarcolemma associated protein b	3	5.57%
	comp55326_c0_seq1	keratin 18	3	3.41%
	comp60031_c1_seq1	heat shock cognate 70 kDa protein	3	4.16%
	comp47208_c0_seq1	actin, cytoplasmic 2	11	32.00%
	comp31865_c0_seq1	elongation factor 1-alpha	5	9.74%
	comp64276_c0_seq5	eukaryotic translation elongation factor 1 alpha 1-like	5	13.28%
	comp56057_c0_seq1	keratin 8, partial	6	5.89%
	comp31760_c0_seq1	ADP/ATP translocase 2	5	11.38%
	comp70485_c0_seq1	nesprin-1 isoform X1	4	0.81%
	comp70712_c0_seq1	cytochrome c	4	32.69%
	comp48176_c0_seq1	Danio rerio zgc:153867, mRNA (cDNA clone IMAGE:7266049), partial cds	3	12.30%
	comp64240_c0_seq1	Histone H2A type 1	3	29.13%

Table S2

Primer sequences and their designated applications.

Gene name	Application	Primer name	Primer sequence (5’-3’)
VP56	pVP56-GFP	VF339	TGAACCGTCAGATCGGGTACCTATGGCCACTCGTGACAGC
		VR342	GGTGGATCCAAGCTTGGGCCCACGTACTTACAGCAAATTACCGTCC
	pVP56-Flag	VF339	TGAACCGTCAGATCGGGTACCTATGGCCACTCGTGACAGC
		VR340	GGTGGATCCAAGCTTGGGCCCACCTTATCGTCGTCATCCTTGTAATC
			GTACTTACAGCAAATTACCGTCC
	pVP56-RFP	VF393	AATTCTGCAGTCGACGGTACCATGGCCACTCGTGACAGC
		VR394	TTATCTAGATCCGGTGGATCCTTACTTACAGCAAATTACCGTCC
	pMC156-VP56	VF583	GCATGGACGAGCTGTACAAGGGCGGCGGCGGCTCAGGCGGCGGCG
			GCTCAGGCGGCGGCGGCTCAATGGCCACTCGTGACAGCC
		VR584	CGCGGTACCGTCGACTGCAGTTACTTATCGTCGTCATCCTTGTAATC
			CTTACAGCAAATTACCGTCCAA
	qRT-PCR	VF73	AGCAGGCTATTCATCACCAGT
		VR74	GTTCTAACGCTCACCGTCTTTTC
VP4	pVP4-Flag	VF347	TGAACCGTCAGATCGGGTACCATAGCGTGGAGACCGACTT
		VR348	GGTGGATCCAAGCTTGGGCCCACCTTATCGTCGTCATCCTTGTAATC
			CACGACGTAAGACGGAGG
	pVP4-GFP	VF347	TGAACCGTCAGATCGGGTACCATAGCGTGGAGACCGACTT
		VR349	GGTGGATCCAAGCTTGGGCCCACCACGACGTAAGACGGAGG
	qRT-PCR	VF146	CGAAAACCTACCAGTGGATAATG
		VR147	CCAGCTAATACGCCAACGAC
RIG-I	pRIG-I-MN155	RF585	TAGGATCTCGAGCTCAAGCTTATGTACGAGCTGGAAAAGGAGAA
		RR586	GTACCGTCGACTGCAGAATTCACTGAGCCGCCGCCGCCTGAGCCGC
			CGCCGCCTGAGCCGCCGCCGCCAGCGTAGTCTGGGACGTCGTATGG
			GTAGTCTCTCAGCGGCCATGTTT
	qRT-PCR	RF230	ACTACACTGAACACCTGCGGAA
		RR231	GCATCTTTAGTGCGGGCG
EF1α	qRT-PCR	EF125	CGCCAGTGTTGCCTTCGT
		ER126	CGCTCAATCTTCCATCCCTT
VP1	qRT-PCR	VF71	GCAATACGCCTCTACTTACTGTTCT
		VR72	ATCGCTTTCTCCACCTCGTCT
NS38	qRT-PCR	VF75	TCTGCTCCGCTTAGAAATGACTC
		VR76	GACGTGGGACAATATGACAACCT
VP35	qRT-PCR	VF77	AATGTCAATTCCACCACCCC
		VR78	CCTTCAGATTCACTATTCCCTCC
IPS-1	qRT-PCR	IF217	GACCGTAAGAAGTCAGCCTCC
		IR218	CCTGAATAACTCTTGATAGCCCTC
STING	qRT-PCR	SF79	TCTTATGCTGGTGTTTGCGTG
		SR80	CTTTGCCTTGAATGAACGAGC
TBK1	qRT-PCR	TF927	CCAGGAGAAATGTTGGGGC

		TR928	TGTAGATGTGGTGGAGTGTCGC
IRF3	qRT-PCR	IF960	ACTTCAGCAGTTTAGCATTCCC
		IR961	GCAGCATCGTTCTTGTTGTCA
IFN1	qRT-PCR	IF590	AAGCAACGAGTCTTTGAGCCT
		IR591A	GCGTCCTGGAAATGACACCT
IFN3	qRT-PCR	IF435	TACATTTATAGAGACTGCGGGTGG
		IR357	TGGAGTGTCTGGTAAACAGCCTT
IFNγ2	qRT-PCR	WF79	CAGCGAACACCTGAAACTAACA
		WR80	CCATCCCAAAGTCATCAAACAT
NF-κB1	qRT-PCR	NF610	CCAGGTGCGGTTTTATGAAGATGA
		NR611	ATGGCTTGGGTTCGCTCGTTT
IκBα	qRT-PCR	IF89	TCCACGAGGCGGAAGATTAT
		IR90	TCTGTGATGACGGCGAGATG
